# The native potato, a symbol of macho expression in the Quechua culture of Peru

**DOI:** 10.3389/fsoc.2023.1268445

**Published:** 2024-01-05

**Authors:** Edgar Gutiérrez-Gómez, Ketty Marilú Moscoso-Paucarchuco, Diana Luján-Pérez, Jaime Carmelo Aspur-Barrientos, Eugenia Rocío Quispe-Medina

**Affiliations:** ^1^Facultad de Ingeniería y Gestión, Escuela de Administración de Turismo Sostenible y Hotelería, Universidad Nacional Autónoma de Huanta, Huanta, Peru; ^2^Universidad Nacional Autónoma Altoandina de Tarma, Junín, Peru; ^3^Facultad de Ingeniería de Minas Geología y Civil.-Facultad de Ciencias Agrarias Universidad Nacional de San Cristóbal de Huamanga, Ayacucho, Peru

**Keywords:** native potato, machismo, Andes, Quechua, ancestry

## Abstract

The present field research focuses on the native potato varieties, *Wira pasña* and *Llumchuy waqachi*, cultivated in the Peruvian highlands at roughly 4,000 m above sea level. The objective is to analyze the macho essence of the names assigned to the native potato, names that represent the social manifestation of women in Quechua culture. Participant observation and interviews about the different daily activities of the participants facilitated the research on the macho essence of the names of the native potato in the Quechua culture. The preponderant Quechua oral sources in the Peruvian Andes did not allow us to identify exactly how names associated with the macho way of social life were assigned to the native potatoes.

## 1 Introduction

The Quechua culture of Peru has a millenary history. The potato tuber (originally from Peru) represents a deeply rooted aspect of the Peruvian identity. Quechua culture is understood in the current context as an identity that is “‘pure indigenous’ or with a peasant lifestyle, they do not try to give any ‘performance’ of their cultural heritage, but this does not mean that the Peruvian people do not expect something different from them” (Renker, 2014, p. 18). The Quechua culture in Peru suffers racial isolation and is a victim of constant discrimination. According to 2017 survey reports, “the main reasons for discrimination are income level (32%), dress (25%), way of speaking (26%), physical features (21%) and skin color (19%)” (Ministerio de Cultura, 2023). Racism is a social phenomenon in Peru that has different forms of manifestation. The Quechua people are also understood as a “large and diverse group of long-standing Andean populations whose mother tongue is Quechua, in its different varieties” (Guerrero and Román, 2019, p. 3).

The native potato, a tuber native to the Peruvian Andes, has a millenary history forged in the microclimates of the Peruvian territory. It is “originally from South America, precisely from the Andes and Upper Peru, its cultivation in other parts of the world was an achievement that required much effort” (Datos LR, 2022). This work does not investigate its origin, agricultural aspect, and survival over many generations. The present research focuses on the analysis of the names assigned to two native potato varieties, *Wira pasña* (good body of a young woman) and *Llumchuy waqachi* (the one that makes the daughter-in-law cry). The subtle sexist manifestations involved in the names are identified. The potato tuber “is innocuous, healthy, natural, and, in addition, it can be consumed with cascara. Within this group with more than 7,000 years of antiquity” (Economía LR, 2021). The names of the different varieties of the native potato in Peru represent the perspective of the Andean man regarding its consumption. Feminine names in Quechua were given to the varieties of potato: “We farmers always say that on the table everything can be missing, except a boiled potato” (Ugarte, 2021).

In Peru, in 2005, Supreme Resolution No. 009-2005-AG. declared every May 30 as National Potato Day. Similarly, in 2021, it was stated that “the World Food and Agriculture Organization (FAO) will declare May 30 as World Potato Day, coinciding with the celebration that takes place in Peru” (Peruano, 2021). This is an official recognition by the Peruvian state for the farmers’ efforts to keep the potato crop and its native variants alive. The national and world consumer of the native potato has no awareness of the macho manifestation of the nomenclatures of the native potato varieties, such as the *Wira Pasña* and *Llumchuy waqachi*: “In Latin America, the highest incidence is in the Andean region. In terms of intimate partner violence, the average in the Andean region is 40.63%, with variations from 34.8 to 46.5%” (BBCMundo.com, 2013). The Quechua meaning of the words in the names is as follows: *Wira* (fat) *Pasña* (derogatory name for young woman, slut), *Llumchuy* (daughter-in-law), *waqachi* (make cry). The present research was conducted in the district of Tambo, Province of La Mar, Ayacucho-Perú Region, Andean highlands in Peru at 3,319 m above sea level. According to the 2017 census, the area had 10,173 inhabitants.

The essential characteristic of the Quechua culture is oral communication, transmitted from one generation to another. The official language is Runasimi (the Quechua language spoken in the area). The millenary history of the native potato varieties is well known; there are “more than 4,000 varieties of this tuber. It was domesticated for the first time in national territory, north of Lake Titicaca, about eight thousand years ago” (PromPerú, 2022). Thousands of native varieties of the native potato have names assigned to them in the Quechua language:

… It is based on direct morphological references (*suytu* = long) or indirect (*wamanpa uman* = hawk’s head). The indirect or metaphorical nomenclature is based on an inherent symbolism that can refer to people (*pasña* = lady), animals (*kuchipa qallun* = pig’s tongue) or related to them (*yutupa runtun* = partridge’s egg), to their possible places of origin (*yana tarmeña* = black tarmeña), and to very particular characteristics (*yuraq llumchuy waqachi* = which makes the white daughter-in-law cry). (CIP, 2006, p. 16)

This research takes into account the following native potato varieties: *Wira pasña* and *Llumchuy waqachi*, among the more than 4,000 varieties that exist to date. The interest of this research is in the sexist nomenclatures and the macho behavior of Andean men, which persists in the high Andean communities. The fecundity of the potato is evident in the Peruvian Andes: “They produce flowers and berries that contain between 100 to 4,000 botanical seeds, and have the quality of being able to be planted from sea level to 4,700 meters of altitude” (PromPerú, 2017). The feminine name assigned to the potato conveys a sense of the macho behavior prevalent in Quechua culture: “it is incoherent that more indigenous women are punished by machismo and violence despite the fact that they are the ones who preserve the ancestral (and therefore cultural) knowledge of the native peoples.” Evidence of this machista cultural phenomenon is exported worldwide to places where the literal meaning of the names of the varieties is not known. Around 16% of its annual production “is native, and around 500 tons are exported in frozen precooked presentations and chips, the United States, Spain, and Germany being the main buyers. There has also been an effort to enter the Asian market, especially China” (PromPerú, 2017). The purpose of this research is to determine whether there has been a generational change in the original macho sense of the names for the native potato that is consumed throughout the world.

## 2 Methodology

This study involved field research in the form of interviews, observation, participant observation, and a field diary. The aim of the research relates to the researchers’ command of the Quechua language and their awareness of comments about native potatoes with striking names. “The indirect or metaphorical nomenclature is based on an inherent symbolism that can refer to people and animals” (CIP, 2006, p. 16). The origin of this study was the researcher’s attendance at different events for National Potato Day, a day of special celebrations every May 30 in different parts of the Peruvian Andes. It is ethnographic research for which constant trips are made and living with them in their daily activities at an altitude of 4,100 meters above sea level. For the interview and participant observation, the researchers are able to speak Quechua, which is the official language of communication in the community. Participating with them in their work activities is fundamental with a guide who is a villager, attending their national events of exhibitions in fairs organized by the authorities of different municipalities that are constant.

In Andean communities, the words *Wira pasña* and *Llumchuy waqachi* are pejorative. They relate to the macho social practices of daily life in Quechua culture. The festivities for the National Potato Day are a special reason behind the research, as are the survival of the Sunday fairs where the barter (exchange of products for another) system of the Inca culture is still in use. In the weekly fairs of the Tambo-La Mar district, farmers from different ecological levels gather to offer their native potatoes in exchange for other products from the La Mar Valley. This is a propitious scenario for research on the macho symbolism of *Wira pasña* and *Llumchuy waqachi* in the Andean communities. Mayoral Resolution No. 114-2023-MDT-LM/A, Article One, recognizes the organizing committee of the “XXVII Regional, Agricultural, Agro-industrial, Handicraft, Folkloric Fair of the Native Potato and XV Festival of the Kuchicanka-Tambo 2023” to be held from June 20 to 24, 2023. The data collection phase began with an interview and the written authorization of the Tambo-La Mar district Municipal Manager, Néstor Choquellana Palomino.

### 2.1 The native potato Wira Pasña as a macho expression in the Quechua culture

In 2023, the district of Tambo celebrated 198 years since its foundation. As part of the celebrations, the deputy tourism manger displayed samples of the native potato varieties in his office. In an interview with the authorities involved in the conservation of the native potato, those involved expressed the ancestral importance of the names and seemed envious of the macho meaning of the name of the *Wira pasña* variety, indicating that the attitude implied comes from the ancestors. It is currently well known in Peruvian cuisine: “so that it is often served with a *Runtus*, a potato stuffed with shrimp with *Wira Pasña*; or as *Leona* accompanying a roasted loin or *Sangre de Toro* as part of a paiche bouillabaisse” (Gestión, 2012). The word *Wira* (adipose fat) in Quechua means fat, fat in humans and animals. *Pasña* is a pejorative vulgar word in Quechua. It means a whore, prostitute, or a woman despised by society: “the Andean cosmovision that have camouflaged discrimination or gender violence: “The idea of seeing the world as a duality made us believe that without a partner we are nothing; or there is the saying ‘the more you hit me, the more I love you” (Gallegos, 2022). It is a popular opinion in the Andes of Peru about the marriage relationship usually agreed upon by the parents. Another variant of this research is the request of hand known in the Andean world as “*Warmi urquy*” which has similar rituals of the potato test *llumchuy waqachi*.

**Figure 1:**
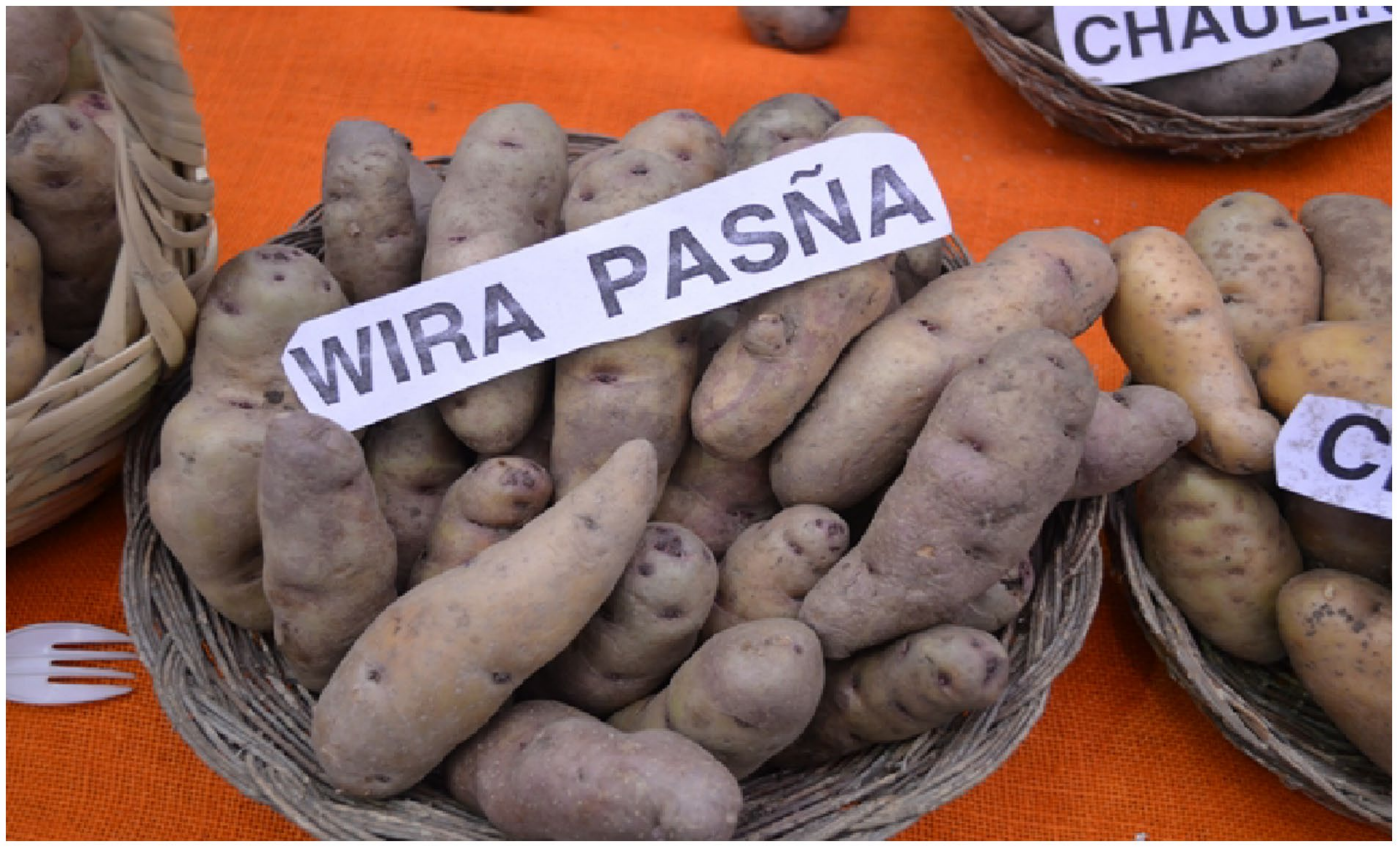
Image of the native potato *Wira pasña*. Source: Ministry of Environment. Uploaded on September 12, 2011. Taken on September 10, 2011. https://acortar.link/3sKnoM.

Quechua culture is purely oral. It is transmitted from one generation to another through orality to maintain its historical past, in this case, the taxonomy of the native potato. It is not known exactly when the description of a young Andean woman with a good body, described by many men as a *Wira pasña*, was assigned as a name to the millenary native potato. This naming is a “demonstration of the way of life of the popular protagonists of the Andean world, who apart from exhibiting their machismo molded with a set of violent acts against women, also show their exquisite mocking humor, sarcastic, ruffian, essentially” (Berrocal, 2022, p. 56). There is neither a Quechua dictionary nor a nationally recognized academy of Quechua that regulates the terms of use of the Quechua language. The relationship between the woman, *Wira pasña*, and the name assigned to the native potato does not have an exact origin. However, they coincide in their history. “Native potatoes such as *wira pasña* and yellow tolerate low temperatures and it is these varieties that are preferred by farmers until they manage to create a potato that completely resists frost” (Redacción, 2014). According to the farmers interviewed, the native potato *Wira pasña* is quite sandy, easy to cook, easy to peel, pleasant in appearance, and durable over time (Figure 1).

In the Quechua culture of the Peruvian Andes, there are several versions of the *Wira pasña*, as corroborated by those interviewed in the community of Usmay, 4,039 meters above sea level, and the Wisca, 3,940 meters above sea level. The name is seen as an expression of flirtation with and harassment of women: “the peculiar humor colludes with the common machismo of current Andean societies” (Berrocal, 2022, p. 53). Expressions such as *añallau Wira pasña* (what a rich woman with a good body), *mikuruyman wak wira pasñata* (I would like to have sex with that woman with a good body), *allim wira pasñam wak warmiqa* (That woman has a good body), *wak llaqtapiqa allin wira pasañakunam kam* (in that village there are women with good bodies), and *Wira pasña* (young woman with a good body) are common.

These expressions were corroborated in the interviews and in observations in the field research field. It was clear that the current culture tries to justify sexism in the Peruvian Andes: “essentialist feminism sees indigenous patriarchy as the only thing responsible for the situation of indigenous women; or, indigenous essentialism justifies machismo and sexism as exclusive products of the colony” (Espinosa et al., 1995). The consumers of the native potato, worldwide, have no awareness of the sexist nature of the names of potato varieties, especially of the *Wira pasña* in the Quechua culture of Peru. There is the idea that the woman herself must fulfill the feminine roles because of her weak nature in the field, the male is in the condition of working for the family’s support: “There was a popular idea in the 1980s about being a woman and empowering women in a way that was almost essentialist: about women’s special powers derived from giving birth, and so on….” (Castedo, 2018).

The feminine accusations of Quechua-speaking women are not limited to the pejorative sexist notion that the *Wira pasña* woman is young, attractive to single or married men. They use expressions such as *qarricha pasña* (young woman whore), *chinakuchi qina pasña* (woman resembling a sow in jealousy), and *qari qatikachaq pasña* (woman who follows men). The correct term used for young women in Quechua culture is *sipas* (young woman). It is the correct expression used by peasants in their daily lives. The native potato variety *Wira pasña* spreads its macho essence in international society due to the emphasis of the state and the potato producers of the Peruvian Andes, who prefer to sell a blurred image of their macho reality, for example: “By scanning the QR code of the packaging you will be able to access the traceability information of this native potato variety, the location of the plot where it was grown, and the name of the farmer” (Redacción, 2021). The process of the industrialization of the native potato, as the QR code shows, does not highlight its macho cultural manifestations in the Quechua culture. When the potato is from previous harvests or has a long duration, it is called *paya papa* (old potato), meaning old woman (longevity).

In the same nomenclature of the native potato in relation to the *Pasña* (vilified young women), there are other variants, such as those set out in the following: “the *Pasña* group is characterized by having compressed tubers with semi-deep eyes. Within the group, varieties are distinguished by a combination of skin colors: *Puka Ñawi Pasña, Azul Ñawi Pasña, Puka Chiqchi Pasña, Yuraq Pasña*, etc.” (CIP, 2006, p. 16). The names assigned to the potato variety are directly related to the feminine characteristics of young women, making them a symbol of sexual harassment in Quechua culture. In the Quechua culture of southern Peru, machismo is still practiced, as evidenced in the following: “According to the results, differences by nationality in machismo could be due to stereotypical patterns of socialization of upbringing that persist in Peruvian culture, rooted in the functioning of its multiple traditions and cultural legacy” (Uezen et al., 2022, p. 200). The tradition persists whereby *pasña* is a contemptuous, macho, Andean Peruvian expression given as a name to the native potato variety *Wira pasña* and its sub-variants, *Puka ñawi pasña* (red-eyed whore woman), *Azul ñawi pasña* (blue-eyed whore woman), *Puka chiqchi pasña* (multi-colored whore woman), and *Yuraq pasña* (white-colored whore woman).

## 3 Everyday life and the native potato *Llumchuy waqachi* in the Quechua culture

**Figure 2:**
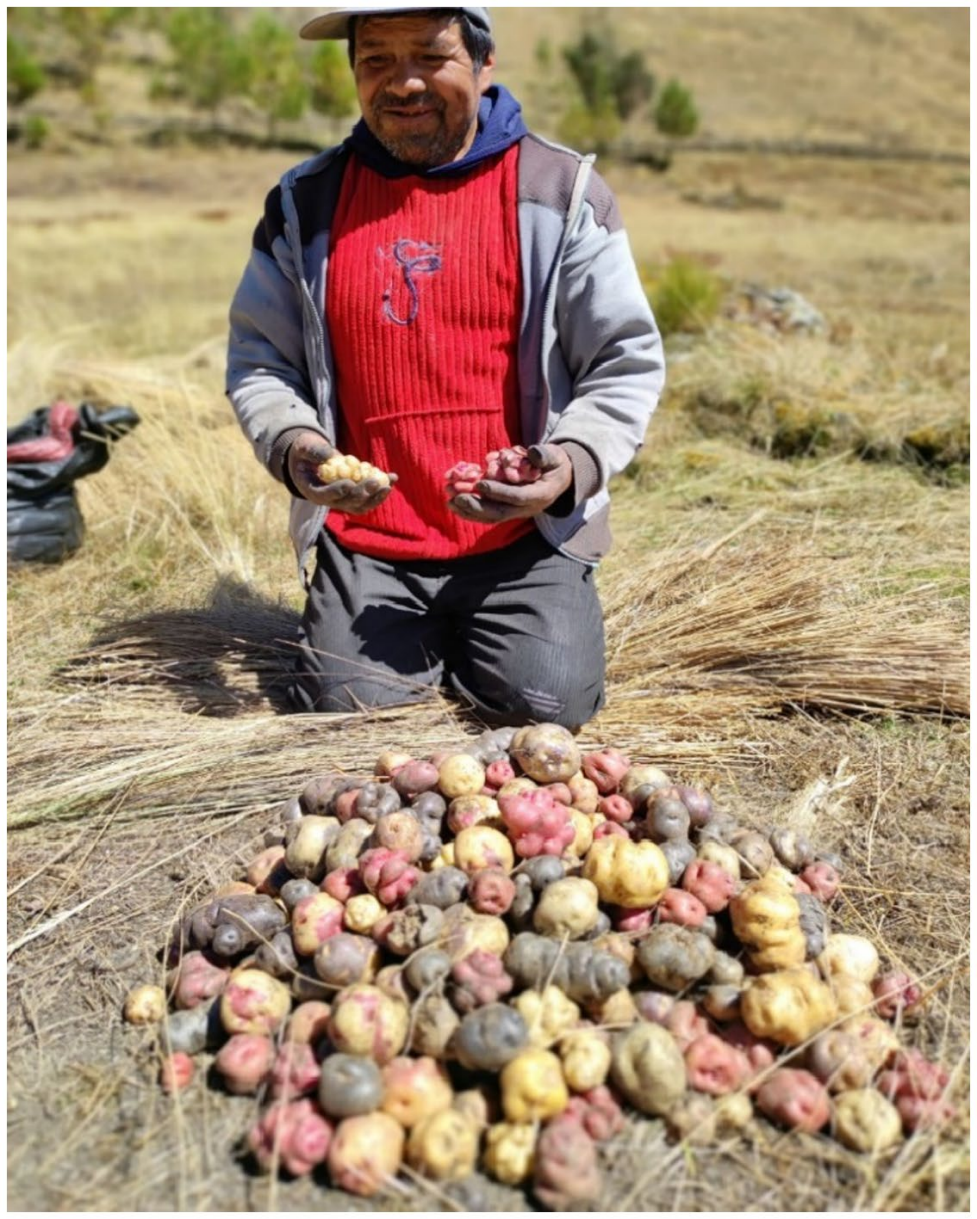
Cirilo Leche with a harvest of *Llumchuy waqachi* potatoes and other varieties in Wiska, Tambo-La Mar, at 3,940 m above sea level. Source: Authors’ photograph (July 2023).

To reach Andean Quechua communities in Peru, a local guide with a direct relationship with the villagers is needed. The farmers are wary of receiving visitors from outside their community without prior authorization. The terror of the internal war between the Shining Path and the Armed Forces in the 1980s and 1990s still lingers. Communication and interviews are conducted in the Quechua language. Cirilo Leche, who lives in the community of Wiska at 3,940 meters above sea level, has cultivated 80 varieties of native potatoes. He knows the traditional information about the feminine names assigned to the potatoes (Figure 2).

The Quechua-speaking community members interviewed from southern Peru agree that the feminine names of native potatoes were assigned by their grandparents (considered Andean settlers, originally from Peru with certain longevity) who perished due to some natural phenomenon. They had their original cosmovision of *paqarina*, outlined as follows:

By *paqarina*, we mean “point of origin or birth” from which an ayllu or community descends. This usually is the mythical place from which men have emerged into the world from underground. This can be a cave, mountain, water source, lake, or feet of trees, and sometimes of an animal. (Bravo, 2015, p. 238)

**Figure 3:**
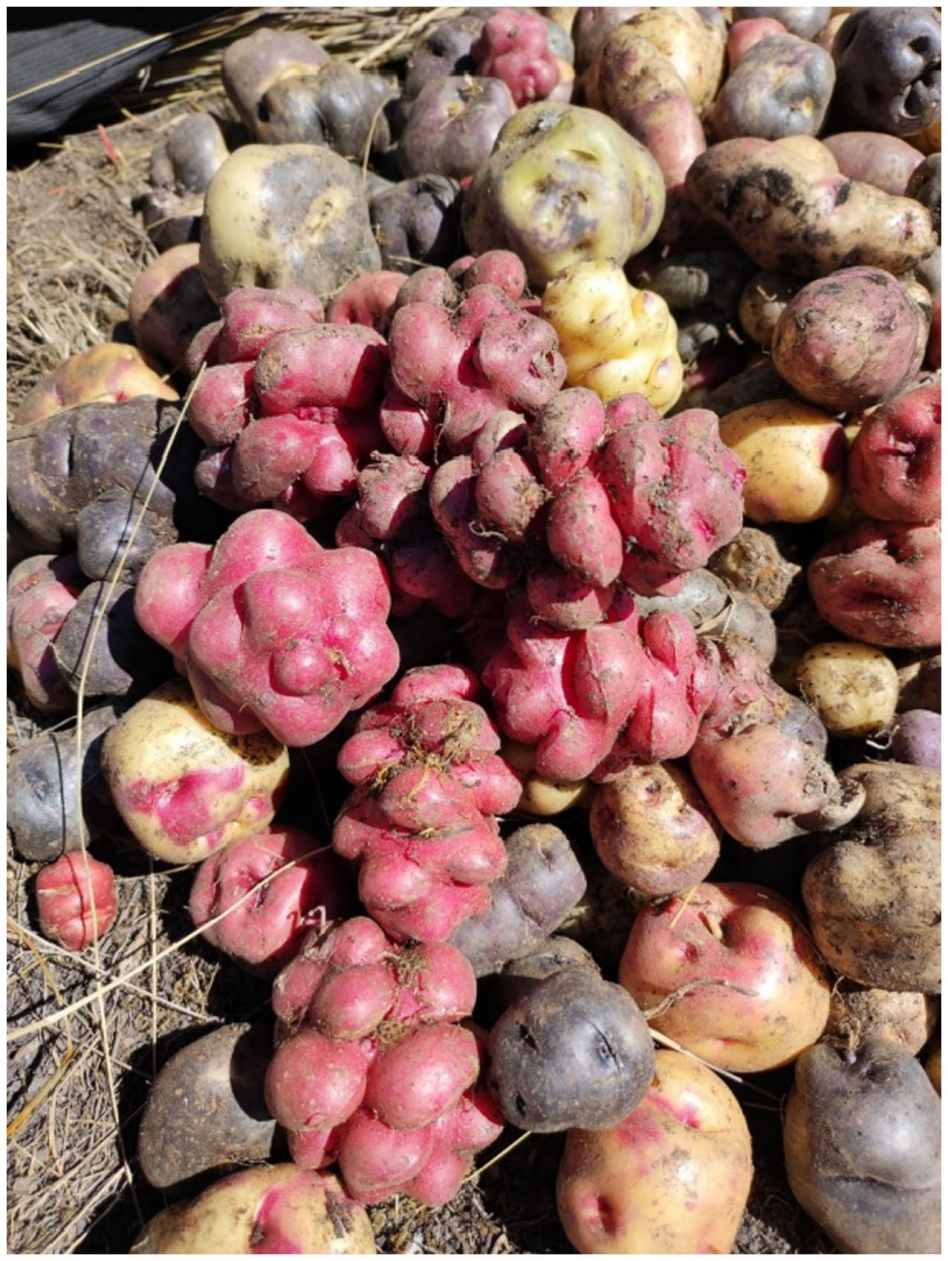
Papa *Puka llumchuy waqachi* (red potato that makes the daughter-in-law cry), recently harvested along with other varieties of *Cirilo Leche* in Wiska, Tambo-La Mar, at 3,940 m above sea level. Source: Authors’ photograph (July 2023).

The *Llumchuy waqachi* is a potato variety that is cultivated to this day. This potato was used to test the culinary skills of the future wife of the son in the Quechua culture (Figure 3). It has a strange peculiarity, outlined as follows:

For this, the daughter-in-law has to peel a potato with a knife, but as the mother-in-law never makes it easy, this is not a “smooth” potato. It is a *llumchuy* full of little eyes and bumps. If the girl’s hand trembles, she has a difficult task, because she must not break the peel. If she does not succeed, she will fail the mother-in-law love test; she is not ready to be a wife and mother. (Galvez, 2015)

In the Quechua culture, a future wife must pass the test of peeling the *Llumchuy waqachi* potato variety in a short time. The women interviewed said that they cried when they could not perform this task. It is essential for the woman to know how to do the household chores and serve her future husband. The woman was told to “peel the potato, not with a knife, but with a llama bone; then, if she peeled well, she was accepted by the family so she could get married. Otherwise, she was returned to her family and that was a dishonor for her family” (Andina, 2022). Those who suffered and cried the most were the brides from low-altitude places with no native potatoes. They were asked to peel *Yuraq llumchuy waqachi* potato (the one that makes the white daughter-in-law cry). The marriage system in the Andean culture was macho. The woman was valued for her ability to perform domestic chores. As a pre-marriage test, some women had to peel a rare potato that some farmers call the *pirca del Inca* (stone walls of the Inca culture). Other current names for this variety are the *ojerona* (it has several eyes) and the *piña papa* (pineapple-like potato).

In interviews, conversations, and observations in the community and in the native potato festivals, it is often said that the practice of testing women for marriage should return. Currently, it is not practiced much. Now, it is suggested that it should be reevaluated because the daughters-in-law of today take no notice of their mothers-in-law. They even beat them and mistreat them, because they have no respect for them, and they do not know how to do the kitchen chores. This is what some of the interviewees indicated when they felt comfortable about the data collection. The men organize *Llumchuy waqachi* potato-peeling contest festivals as a symbol of the continuous subjugation of women, of the prevalent machismo in the Quechua culture: “We are doing it to recover our history, our culture. In rural villages, it was used to test the future daughter-in-law,’ said Raul Ccanto Retamozo, a member of the Association of Guardians of the Native Potato (Aguapan) *Junín*” (Andina, 2022). Quechua-speaking women celebrate the extinction of this ancestral macho practice because it made them cry and feel ashamed in front of the community when they failed the test and were judged not to be ready for marriage.

**Figure 4:**
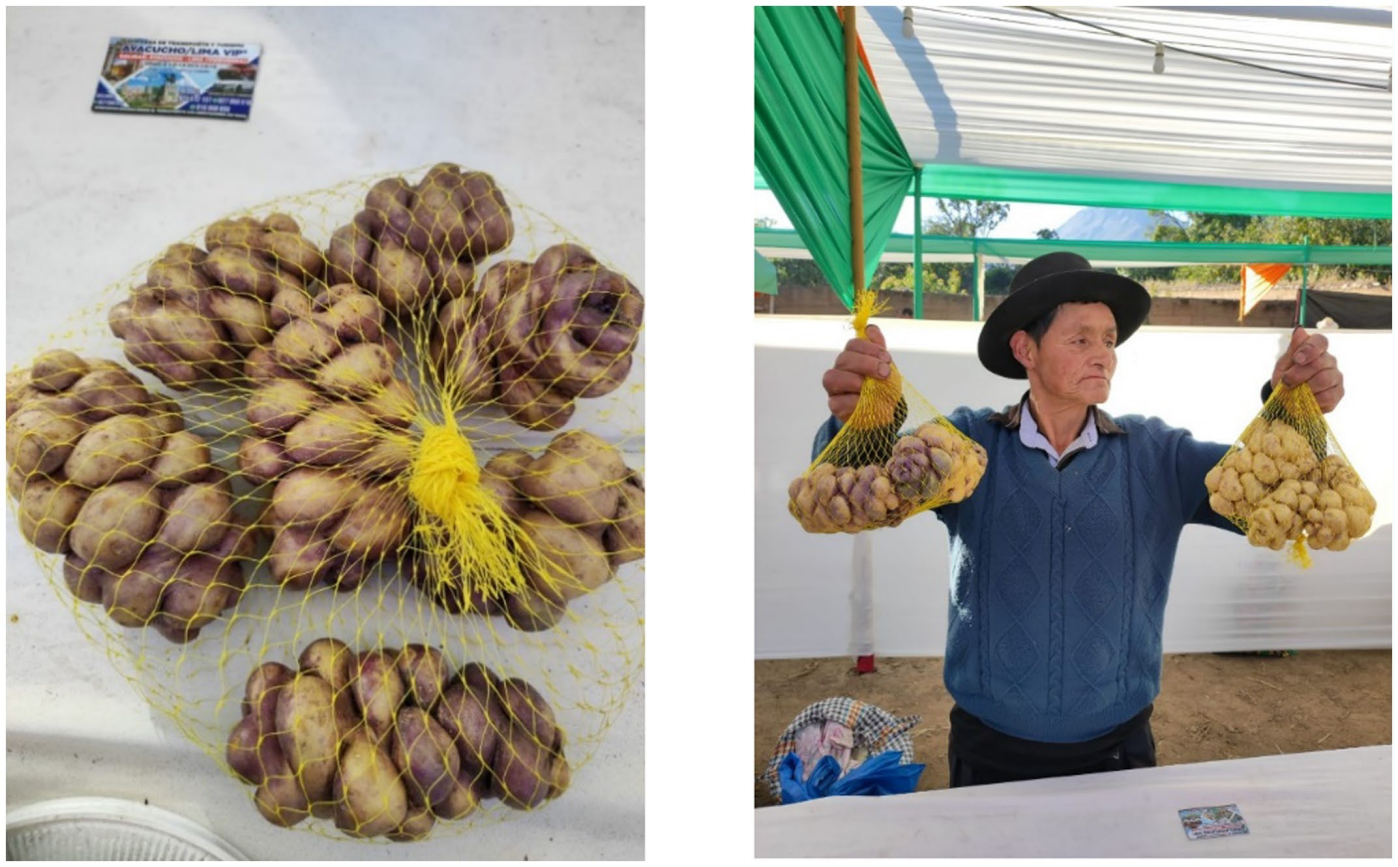
*Yana llumchuy waqachi* potato (black potato that makes the daughter-in-law cry) exhibited by a producer at the “XIX Custard Apple Festival X Avocado Festival EXPO 2023” Ninabamba-VRAEM July 10–14, at an altitude of 2,331 m. Source: Authors’ photograph (July 2023).

The remoteness of the district, provincial, and departmental capitals means that Quechua communities have little access to global information on gender equity. Household chores are only performed by women. The labor force in the Peruvian Andes consists of men. The women interviewed are enthusiastic about the practice of *Llumchuy waqachi* being gradually replaced by equity in domestic chores. The importance given by the provincial, district, and town municipalities to the festivals in their jurisdiction encourages the promotion of the native potato, with its macho names. These products were exhibited at events such as the “XIX Custard Apple Festival X Avocado Festival EXPO 2023” in Ninabamba-VRAEM from July 10 to 14 (Figure 4).

Figure 4 shows the physical characteristics of the native potato *Llumchuy waqachi*, which has strange shapes. They look like several potatoes in one, which is not the case, but, according to the farmers, they have eyes. The visitors to the fairs that are organized on the anniversaries of the districts, provinces, and populated centers, for example, National Potato Day, are aware of the meaning of the name of the *Llumchuy waqachi* potato, and that this was a macho expression in its ancestral origin. In the visits made as part of the data collection, other forms of macho manifestations with markedly macho characteristics were evidenced, such as the *Warmi urquy* (asking for the hand of a woman in marriage) and the acllay of wheat (collecting wheat from the sand after the harvest) that occurs in Andean communities between 2,800, and 3,200 meters above sea level. Another variable of the research is the *Warmi urquy* and acllay (select) of wheat of macho essence.

The ancestral culture does not reflect everything positive, and some macho social practices should disappear, as evidenced in the following: “History shows machismo in various forms of social control, overvaluing the male to the detriment of women” (Gutiérrez, 2019, p. 293). The Peruvian state aims to reach the most remote places where marked machismo, accepted by the whole community, continues. There are still people alive who have passed the trial stage to become a future daughter-in-law and wife, for example, María Bartola Lazo Rojas, who is 94 years old. The elderly woman explained how she had to go through this test to be accepted as a daughter-in-law: “She checked and criticized the potato peeling of all the participants” (Apurímac, 2015). In Quechua communities, where information is not yet available, machista practices persist and are approved by the population.

### 3.1 Current context of the *Wira Pasña* and *Llumchuy waqachi* in high Andean communities

The survival of ancestral customs that were once the way of life of Peruvians in the Andes continues to cause interest and controversy for its sexist and vertical manifestation in a society that seeks gender equity. Regardless of its sexist manifestation and the thousands of varieties of the native potato in Peru, the *Wira pasña* and *Llumchuy waqachi* varieties continue to be promoted and cause national and global interest, as in the following: “It is a highly adaptable plant, because it grows under the most diverse climatic and soil conditions, and today feeds, in addition to the 33 million Peruvians, children, and adults around the world” (Perú informa, 2020). In the high Andean Quechua-speaking communities of the Tambo-La Mar district, the native potato is dried and turned into *chuño* (cold-dried potato): “It is prepared in the months of June and July, in the middle of winter in the southern hemisphere, when *el friaje* arrives to the high plateau of the Andes and temperatures begin to drop at night to −−5 degrees” (Rosendo, 2017).

**Figure 5:**
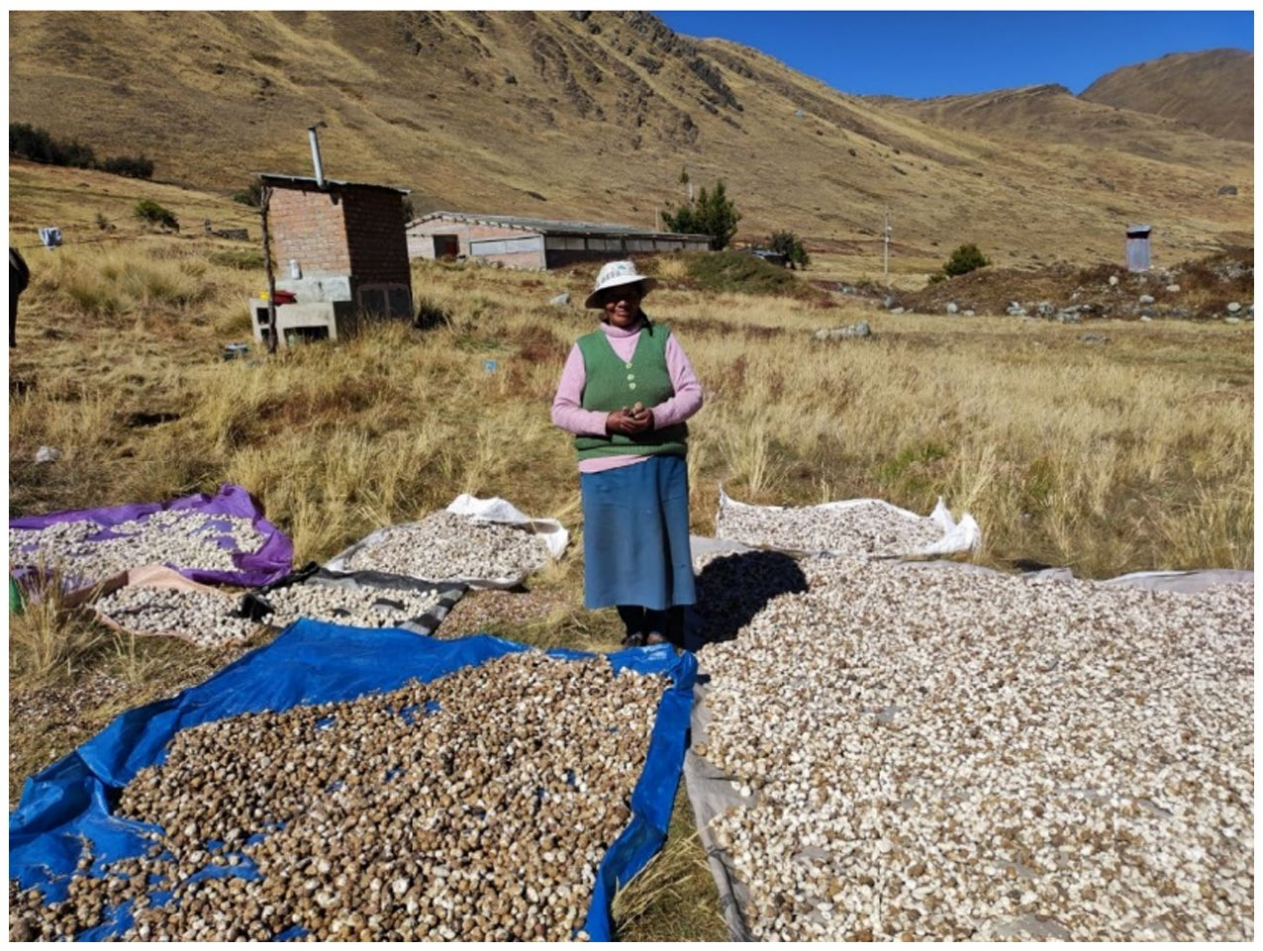
The preparation of *chuño*, *Wira pasña* variety, in the Andean community of Usmay. Source: Authors’ photograph (July 2023).

In the community of Usmay, there is a lagoon called Yanaccocha (black lagoon) whose waters come from Yana Orqo (black hill), each of the two places with its deities that the community worships. The river that comes out of Yanaccocha is used to prepare *chuño*, almost all of its banks, especially the *Wira pasña* variety which is very good for preparing *chuño* that maintains its texture and is white. The product is for the market in the lower parts of the Andes or for barter for another product, as well as for consumption: “In the Andes of Peru at the time of November and December there is no fresh food” (Rosendo, 2017). *Chuño* is an essential food. A generational change of valuing the *Wira pasña* and making the daughter-in-law cry with the *Llumchuy waqachi* is gradually occurring. Female participation is increasingly visible in women’s rights and duties, as stated by the Andean women in the interviews (Figure 5).

**Figure 6:**
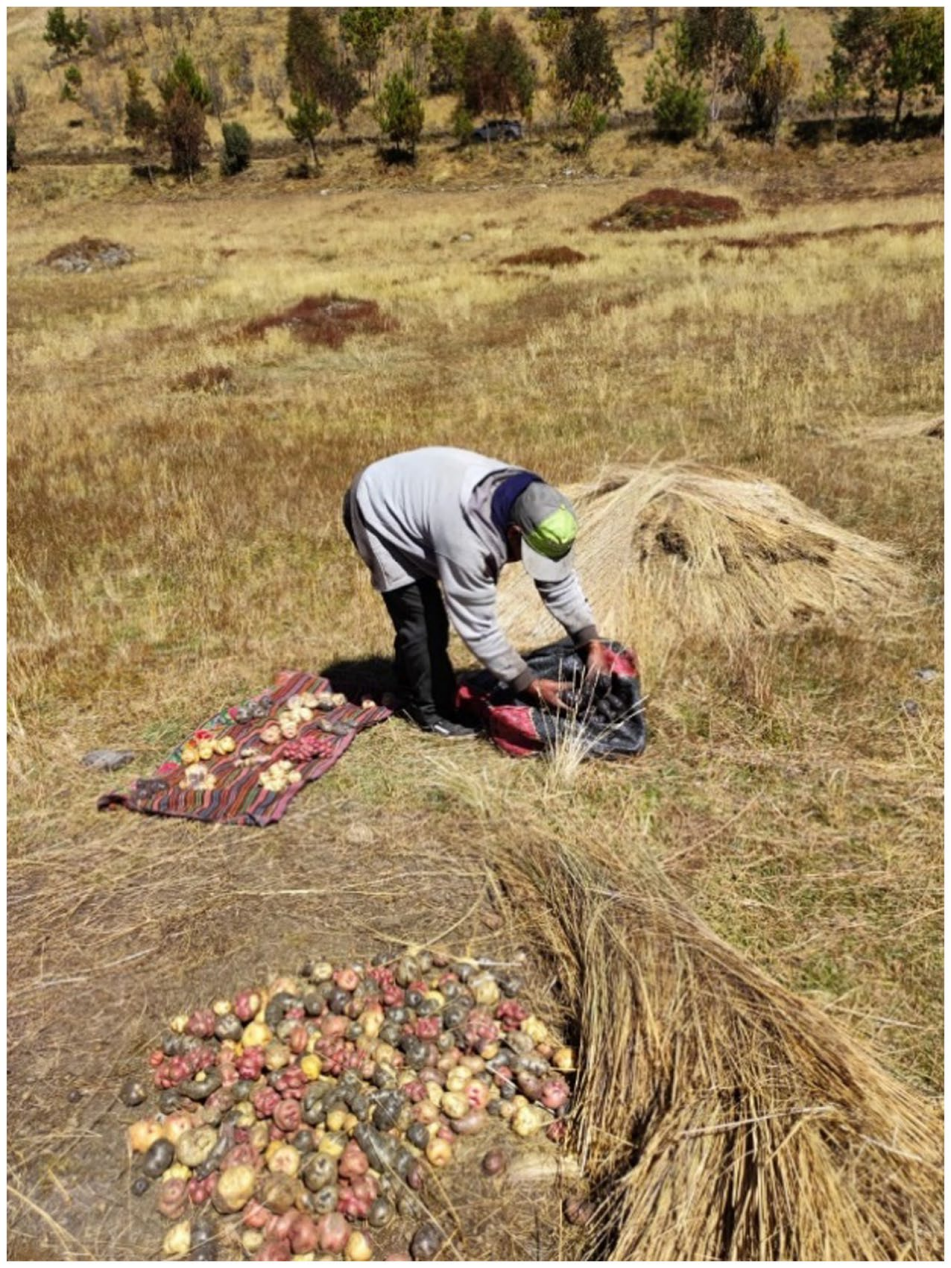
An excited Cirilo Leche displays his potatoes to be photographed for the world to see his production. Due to solar radiation, he has to cover them with *ichu* (natural Andean grass) as he harvests them. Source: Authors’ photograph (July 2023).

The community of Usmay and Wiska, geographically divided by the slopes of the Yana Orqo hill at a distance of approximately two kilometers, has border problems with the San Francisco-La Mar District of the Apurímac, Ene, and Mantaro River Valley (VRAEM) and the Tambo-La Mar district. According to Cirilo Leche, who grows 80 varieties of native potatoes in Wiska, they do not receive any support from the Peruvian government because of the border problems. He indicates that he hears news about economic reactivation bonds and vaccines for COVID-19, but they never received them. They were totally displaced by the Shining Path Movement, which affected “approximately 600,000 people (120,000 families). Slightly more than 80% of these came from the south central region of the country (Ayacucho, Huancavelica, Apurímac and Junín) and 70% of the total came from the rural sector (Andean communities)” (Cordero, 2006). Currently, they have made a gradual return and are dedicated to growing native potatoes, recovering the millenary tradition of the Incas with organic natural fertilizer products: “Their decision to return is firm, they have made many efforts to return, they are willing to face poverty and all the obstacles that arise except political violence” (Cordero, 2006). Today, native potatoes are harvested in a Peruvian Andean community (Figure 6).

Farmers involved in the cultivation of native potatoes want their products to have national and global visibility. They want to experience economic growth and are happy to have people visit their production sites. Moving their products to the market is complicated by the economic cost and the consumer preference for inorganic potatoes produced with chemical fertilizers. Another way they maintain their crops is through the barter system, an “ancestral commercialization, it contributes to the equitable exchange of goods and/or services fundamentally in indigenous and peasant communities” (Artieda et al., 2017, p. 296). During the visits conducted for this research, many farmers indicated that they should bring products from other areas of the valley to exchange them.

### 3.2 Potato festivals in Peru

In Peru, a series of festivals dedicated to agricultural production are held on different dates according to the Andean calendar. This activity takes place in the three natural regions of the Peruvian coast, highlands, and jungle. In the present investigation, attention is dedicated to the National Festival of the native potato in its diverse varieties that have sexist names, especially *Wira pasña* and *Llumchuy waqachi*:

Seven provinces of Apurímac participated in the festival of native potato varieties: *serreñita, camotillo, añil, riti sisa* (snow flower), *yana wayro* (black wayro), *duraznilla, waqrillo* (similar to horns), *llumchuy waqachi* (which makes the daughter-in-law cry who makes it difficult to peel the potato), *wenqos* (crooked); among others. (Galv, 2013)

The native potato festival in the interior of the Peruvian territory encourages the consumption and promotion of the different varieties that currently exist. The awarding of the prize for the best native potato variety promotes the farmers of the Peruvian highlands as producers of authentic organic products. “The ‘T’ikapapa’ was chosen from among 942 proposals from around the world, because it constitutes a business alliance for the sale of native Peruvian potatoes, a social marketing concept that allows high Andean farmers to commercialize native potatoes” (Coloma, 2007). The achievement acquired at the international level gives recognition to the Andean region. The highlands area depends on agriculture because temperatures reach zero degrees in winter. In 2005, the Peruvian government recognized May 30 as National Potato Day, promoting a series of regional, national, and international activities. “The president of the Association of Exporters (Adex), Juan Varilias, announced that the export of native potato increased by 211% in the last five years, becoming one of the Peruvian products with the highest international demand in the market” (Adex, 2016). The international supply and demand for the millenary Andean tuber is growing, competing with other potato producers in the world.

Farmers in the Peruvian Andes prepare for the festival that takes place every year promoted by local, regional, and national governments. The festival is a valuable opportunity to exhibit their best carefully cultivated, organically fertilized native potato products. “In the case of new destinations to explore, Varilias highlighted the potential of the United Arab Emirates and China” (Adex, 2016). This keeps the cultivation of the native potato alive. The local market has a consumer economy, especially with barter (exchange of potato for another product) the Inca way of trading, which still survives in the Andes of Peru. Modern agricultural engineering techniques encourage genetic improvement to acclimatize to low temperatures: “In Peru we have 3,500 varieties of the tuber, while in Cusco about 800 are produced, many of them genetically improved in order to withstand lower temperatures and be cultivated in higher areas” (Sociedad, 2022). Modern research on native potatoes encourages their cultivation on a larger scale, as does chemical and nutritional research by specialists in the field of consumption.

The celebrations and recognition of the native potato at the international level are extensive, promoting its importance in world consumption. In 2013, “even Google wanted to commemorate the ancestral tuber, dedicating its doodle-image that appears on the home page of its search engine on Google Peru Day” (Sociedad, 2013). Because it is the most valued search engine in the world, its promotion of the potato and of Peru makes those who grow native potatoes in Peru proud of their achievements. A negative aspect of the activities of those who rightly promote the preponderance of the native potato and its nutritional components is their lack of awareness that the native names have sexist syntactic roots in the Peruvian Andes that do not detract from its importance in the world market. The Quechua language is an ancient language: “Water and land in the Peruvian Andes have a sacred foundation because they give life and are related to feminine divinities such as Mama Pacha (Mother Land) and Mama Qocha (Mother Lagoon)” (Gutiérrez et al., 2023, p. 14). The meanings of the names persist in the native-speaker context. Therefore, the *Wira pasña* and *Llumchuy waqachi* nomenclatures are essentially sexist in the Quechua culture.

## 4 Discussion

In Peru there are Andean communities that do not have much relationship with the central government, access to information is limited, the homes of the farmers are dispersed, they have little socialization among their members. Meetings are sporadic due to the arrival of some local government representative who wishes to coordinate any help to the community; these meetings are usually attended only by men. This masculinized practice is progressively changing in communities close to cities, where access to information and education is possible: “La República compiled a list of Peruvian programs with large audiences that have been broadcasting sexist, sexist and even racist messages, which encourage violence against women” (Bueno, 2020). Progressive change in gender equality is not positive due to the influence of the media with sexist and macho content, especially for Andean women: “A broader political space is required to discuss and legislate on artistic presentations and, above all, the control of information that discredits formal education in Peru” (Gutiérrez & Munaris, 2023). The control of media information is not effective by the Peruvian State, this form of communication undermines the progressive development towards gender equity in Peru, especially in the field that is the subject of this work. The Andean communities use the ancestral language Quechua in their daily communication; learning the second language, Spanish, generates linguistic interference and this is used to make fun of Quechua speakers.

The gap is still strong towards gender equity, especially in the Peruvian Andes: “Being a woman in Peru is not easy, but if you are also Aymara, Quechua or Shipiba it is even worse. There is a combination of machismo and racism” (Glave, 2023). The machista manifestations are evident in the case of vulnerable people who have native languages as their only form of communication, where machismo still persists, based on this field research. Consequently, the economic gaps of women in the countryside are still dependent on men, because of the same situation that relates to a peasant woman with the potato *wira pasña* woman prepared for physical labor and to be at the service of her husband with the test of peeling the potato *llumchuy huaqachi*. The assertions of the research of the present work are confirmed by other sources on women’s work in Peru: “economic gaps are evident, which is a pending task of all governments in office to establish the minimum economic and social differences, the rural sector is abandoned to the drift of all governments that only seek electoral gain” (Moscoso and Gutiérrez, 2023, p. 266). These behaviors on the part of the Peruvian State, political actors and the media stall the progress of gender equality in the Peruvian Andes.

## 5 Conclusion

The Quechua culture has its own idiosyncrasies, represented especially by the present-day Quechua-speaking Andean communities. This Quechua society has its specific forms of social coexistence. The government has little presence due to its geographical location far from the capital and its inaccessibility to vehicular transport. Andean men live in these social and geographic conditions, hence, the indeterminate origin of the macho name given to the native potato, which is only produced at high altitudes. The interviewees repeatedly concluded that these macho names were given by the original grandfathers of the *paqarinas*, who died of natural causes. Their machista existence is demonstrated by examples such as the “contest of peeling pineapple potatoes for the new daughters-in-law of this district” (Eventos Sociales, 2022).

For a long time, machismo was practiced literally with the nomenclature of the native potato, especially the *Wira pasña* and *Llumchuy waqachi* varieties. The social phenomenon of the internal war in Peru between the Shining Path and the Armed Forces decimated the peasants of the Andes. As a consequence of the social conflict, they were forced to migrate to the cities. They became displaced people living on the periphery of nearby cities. In this new social interrelation, the prevailing machismo in the Quechua culture diminished. Currently, they have become returnees, supported by the Repopulation Support Program (PAR). They have recommended the cultivation of native potatoes. In the interviews and observations carried out in the Tambo-La Mar communities, machismo still exists in the representation of the women men admire, especially in the *Wira Pasña*. The implication is that only a woman with a good body can work in the fields, is destined for good reproduction, and will survive in the Quechua culture. This is complemented by the social culture of making the daughter-in-law cry (*Llumchuy waqachi*). If she does not pass the challenging potato-peeling test, she is not fit for marriage.

The women are happy that the social practice of *Llumchuy waqachi* has been disappearing, although they have changed the ways in which they must learn the essential skills of Andean cooking. The men underhandedly state that they must maintain the practice of making the daughter-in-law cry, because nowadays women know nothing about domestic chores; they even beat their mothers-in-law. The old marriage test is still promoted in special events, for example, “on the occasion of the first anniversary of the district of José María Arguedas, in An-dahuaylas, Apurímac region, a peculiar potato-peeling contest was held among aspiring wives, who demonstrate their skills to their future mothers-in-law” (Apurímac, 2015). This demonstrates the literal practice of the nomenclature of the native potato *Llumchuy waqachi*. The anniversary activity of the municipalities of Quechua Peru generates the macho practice in a potato-peeling contest, “testing the future *yerna* (daughter-in-law)” (Anitapv, 2023).

The machismo of the name of the native potato *Wira pasña* is still in force in Quechua culture. It is the vulgarized manner of appreciating a woman in Quechua. The interviewees evidenced the intact and literal forms of their understanding of social interrelations. The manifestations of these interactions are subtle and underhanded. The women interviewed often laugh in embarrassment at the question in Quechua before giving their answer. Some of them say that women also look for an *Allin Qari* (good man) when they see a *Maqta* (young man) because he is prepared for work in the fields and will not make the future wife lack solvency. They also claim that, in some parts of the Quechua culture, there is a test for men who aspire to be husbands. State intervention is fundamental to eradicating machismo in Quechua culture. A report indicates that research on machismo found the following: “The condition of supposed superiority was recognized by 28% of women and 44.2% of men” (Proyecto de Desarrollo Territorial Sostenible, 2022). Tambo-La Mar district report.

The limitations of the research are its inaccessible condition, low temperatures at night, and high temperatures during the day. Farmers go to their highest farms in the early morning hours. Due to the climatic conditions, only potatoes are produced, and the farmers we interviewed asked for the products they need from the lowlands. Other farmers accused the researchers of making money from the interviews and conversations, demanding financial payment. Knowledge of the Quechua language and working with a guide facilitated the production of this article.

## Data availability statement

The original contributions presented in the study are included in the article/supplementary material, further inquiries can be directed to the corresponding author.

## Ethics statement

Ethical approval was not required for the study involving humans in accordance with the local legislation and institutional requirements. Written informed consent to participate in this study was not required from the participants or the participants’ legal guardians/next of kin in accordance with the national legislation and the institutional requirements. Written informed consent was obtained from the individual(s) for the publication of any potentially identifiable images or data included in this article.

## Author contributions

EG-G: Funding acquisition, Investigation, Project administration, Writing – original draft. KM-P: Methodology, Resources, Visualization, Writing – review & editing. DL-P: Conceptualization, Data curation, Formal analysis, Software, Validation, Writing – original draft. JA-B: Supervision, Writing – original draft, Writing – review & editing. EQ-M: Funding acquisition, Writing – review & editing.
